# Neonatal presentation of a patient with Liddle syndrome, South Africa

**DOI:** 10.4102/ajlm.v12i1.1998

**Published:** 2023-04-14

**Authors:** Nicolene Steyn, Bettina Chale-Matsau, Aron B. Abera, Gertruida van Biljon, Tahir S. Pillay

**Affiliations:** 1 Department of Chemical Pathology, Faculty of Health Sciences, University of Pretoria, Pretoria, South Africa; 2 National Health Laboratory Service, Tshwane Academic Division, Pretoria, South Africa; 3 Inqaba Biotechnical Industries (Pty) Ltd, Pretoria, South Africa; 4 Division of Paediatric Nephrology, Department of Paediatrics, Faculty of Health Sciences, University of Pretoria, Pretoria, South Africa

**Keywords:** Liddle syndrome, epithelial sodium channels, genetic sequencing, hypertension, hyporeninaemia, hypoaldosteronism

## Abstract

**Introduction:**

Liddle syndrome is an autosomal dominantly inherited disorder usually arising from single mutations of the genes that encode for the alpha, beta and gamma epithelial sodium channel (ENaC) subunits. This leads to refractory hypertension, hypokalaemia, metabolic alkalosis, hyporeninaemia and hypoaldosteronism, through over-activation of the ENaC.

**Case presentation:**

We describe a 5-day old neonate who presented with severe hypernatraemic dehydration requiring admission to Steve Biko Academic Hospital in South Africa in 2012. Further evaluation revealed features in keeping with Liddle syndrome. Two compound heterozygous mutations located at different subunits encoding the ENaC were detected following genetic sequencing done in 2020. The severe clinical phenotype observed here could be attributed to the synergistic effect of these known pathological mutations, but may also indicate that one of the other variants detected has hitherto undocumented pathological effects.

**Management and outcome:**

This child’s treatment course was complicated by poor adherence to therapy, requiring numerous admissions over the years. Adequate blood pressure control was achieved only after the addition of amiloride at the end of 2018, which raised the suspicion of an ENaC abnormality.

**Conclusion:**

To our knowledge, this is the first Liddle syndrome case where a combined effect from mutations resulted in severe disease. This highlights the importance of early recognition and management of this highly treatable genetic disease to prevent the grave sequelae associated with long-standing hypertension. Whole exome sequencing may assist in the detection of known mutations, but may also unveil new potentially pathological variants.

**What this study adds:**

This study highlights the importance of developing a high index of suspicion of tubulopathy such as Liddle syndrome for any child presenting with persistent hypertension associated with hypokalaemic metabolic alkalosis.

## Introduction

Liddle syndrome, the most common monogenic cause of hypertension,^1^ is an autosomal dominantly inherited disorder typified by salt-sensitive hypertension, hyporeninaemia, hypoaldosteronism, metabolic alkalosis and variable hypokalaemia.^2^ Even though symptoms and signs may present in infancy, the diagnosis is often significantly delayed.^3^

Sodium reabsorption in the epithelial cells of the distal renal tubule is regulated by the epithelial sodium channel (ENaC).^4^ Liddle syndrome arises from activating mutations of the *SCNN1A, SCNN1B* and *SCNN1G* genes,^5^ which encode for the intracellular carboxy-terminal domains of the alpha, beta and gamma ENaC subunits. This results in an elevated number of channels and markedly increased independent activity with consequent sodium and water retention, hypertension and negative feedback suppression of renin and aldosterone secretion.^2,3^

## Ethical considerations

Written consent was obtained from the child’s parents and ethics approval obtained from the Research and Ethics Committee at the University of Pretoria (No. 536/2020). Confidentiality was ensured in the preparation of this case study.

## Case presentation

A male patient of Ethiopian descent, born in November 2012, presented on day five of life with severe hypernatraemic dehydration and acute renal failure requiring admission. On examination, he appeared severely wasted and dehydrated with absent femoral pulses. Abdominal ultrasound revealed a thrombus in the aorta, attributed to the hyperviscosity associated with the severe dehydration. The thrombus resolved following heparin therapy and the child was discharged upon resolution of his renal failure with rehydration in December 2012.

He was reviewed a week after discharge and found to have hypertension. During subsequent admissions over the years of follow-up, no clotting abnormalities were found and renal ultrasonography revealed no new thrombus nor renal abnormalities. No cardiac abnormalities were detected on sonography. There was no history of consanguinity.

Laboratory results (analysed on an Abbott Architect ci8200 (Abbott Laboratories, Chicago, Illinois, United States) up to 8 years of age revealed potassium values ranging from 2.0 mmol/L to 3.0 mmol/L (reference interval [RI]: 3.7 mmol/L – 5.9 mmol/L) and sodium levels 159 mmol/L – 171 mmol/L (RI: 136 mmol/L – 145 mmol/L) (Table 1). Metabolic alkalosis was also present (HCO_3_^−^ = 29 mmol/L to 34 mmol/L [RI: 23 mmol/L – 29 mmol/L]). Random urine potassium was elevated (12.0 mmol/L – 22.0 mmol/L [RI: < 10 mmol/L]) despite the low serum potassium on presentation.

**TABLE 1:** Laboratory results trends during follow-up at Steve Biko Academic Hospital in South Africa, 2020.

Analyte	Reference ranges	Age								
		At birth Nov 2012	1 day Nov 2012	4 months Mar 2012	8 months Jul 2013	10 months Sept 2013	2 years Jan 2015	4 years Feb 2017	5 years May 2018	8 years Aug 2020
Sodium (mmol/L) *(ISE)*	136–145	171	161	139	141	143	136	135	150	137
Potassium (mmol/L) *(ISE)*	3.7–5.9	5.5	3.1	3.6	2.5	1.7	1.3	6.1	2.4	1.8
Chloride (mmol/L) *(ISE)*	98–113	129	131	107	94	94	99	100	104	99
Bicarbonate (mmol/L) *(ISE)*	23–29	8	19	23	34	37	26	14	34	27
Anion gap (mmol/L) *(calculated)*	9–16	40	17	13	16	14	12	27	-	13
Urea (mmol/L) *(urease)*	0.7–4.6	58.1	29.4	1.4	2.8	2.1	5.5	3.3	1.3	1.78
Creatinine (μmol/L) *(kinetic alkaline picrate)*	14–34	530	182	13	18	19	27	58	39	45
Urine sodium (mmol/L) *(ISE)*	< 10	-	-	42	-	-	69	-	-	-
Urine Potassium (mmol/L) *(ISE)*	< 10	-	-	18	-	-	22	-	-	-

ISE, ion selective electrode.

Initial investigations in 2019, for the specific R563Q mutation and mutations on exon 13 of the beta subunit of the ENaC (most common causes of Liddle syndrome in South Africa), were negative. Further investigation was undertaken, with the aid of external funding, of all exons and exon-intron boundaries of the alpha (*SCNN1A* [GenBank NM_001038.5]), beta (*SCNN1B* [GenBank NM_000336.2]) and gamma (*SCNN1G* [GenBank NM_001039.3]) subunits encoding for the ENaC; these were amplified by polymerase chain reaction and sequenced using the Nimagen, BrilliantDye™ Terminator Cycle Sequencing Kit V3.1, BRD3-100/1000 (NimaGen, Nijmegen, the Netherlands), according to manufacturer’s instructions, in 2020.

**FIGURE 1:**
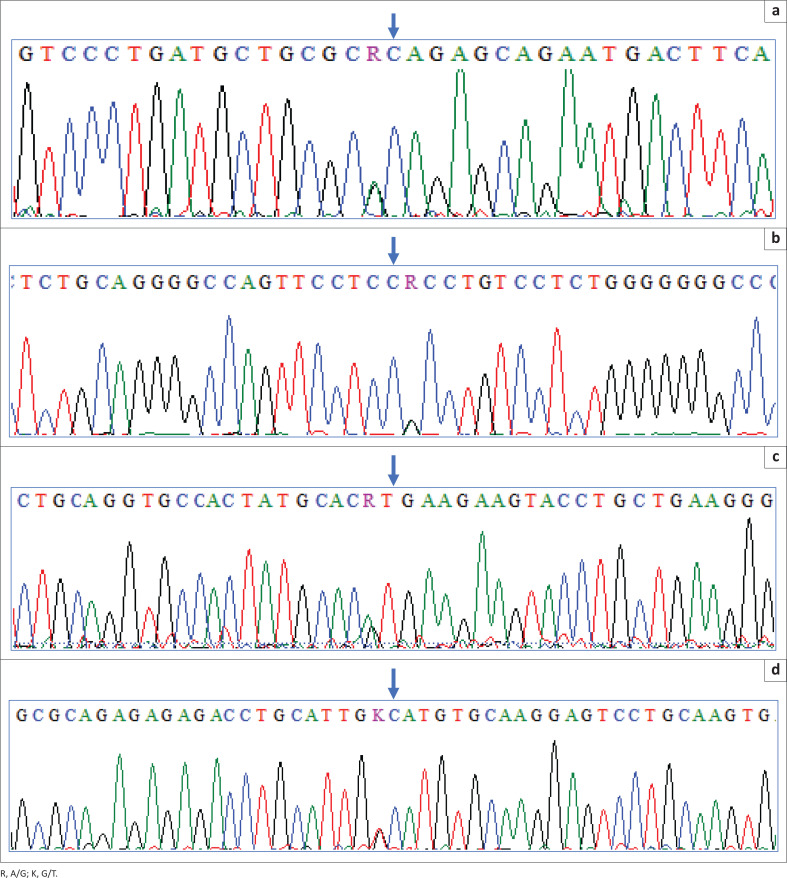
Sanger Sequencing electropherogram results of subunits *SCNN1A* and *SCNN1B* of the ENaC indicating mutations (arrow). (a) Sequence electropherogram showing a heterozygous c.1000G>A mutation (chr12:6355415 [GRCh38.p14]) in exon 5 of *SCNN1A*. (b) Sequence electropherogram showing a heterozygous c.1987A>G mutation (chr12:6347896 [GRCh38.p14]) in exon 13 of *SCNN1A*. (c) Sequence electropherogram showing a heterozygous of *SCNN1A* c.7G>A (chr16:23348606 [GRCh38.p14] in exon 2 of *SCNN1B*. (d) Sequence electropherogram showing a heterozygous c1325G>T mutation (chr16:23377219 [GRCh38.p14]) in exon 9 of *SCNN1B*.

Two sets of compound heterozygous transition mutations were found in the coding regions of the *SCNN1A* and *SCNN1B* genes (Figure 1). In *SCNN1A*, c.1000G>A in exon 5 resulted in Ala334Thr substitution and c.1987A>G in exon 13 led to Thr663Ala amino acid change. In the *SCNN1B* gene, there was a c.7G>A mutation in exon 2 leading to a Val3Met substitution, and a c.1325G>T mutation in exon 9 leading to a Gly442Val substitution. No mutations were detected in the coding region of the *SCNN1G* gene.

## Management and outcomes

The patient was initially managed on multiple antihypertensive drugs and potassium supplementation, but the treatment course was complicated by poor adherence to therapy and follow-up, resulting in numerous re-admissions over the years to achieve blood pressure control. Effective blood pressure control was only achieved on commencement of amiloride in 2018, years after the initial presentation. This prompted the investigations for an ENaC abnormality.

## Discussion

In a neonate presenting with hypernatraemia and hypokalaemic metabolic alkalosis, the use of diuretics, persistent vomiting, nasogastric free drainage losses or a tubular disorder such as Gitelman or Bartter syndrome should be considered. If hypertension is also present, secondary causes such as congenital adrenal hyperplasia, primary hyperaldosteronism, syndrome of apparent mineralocorticoid excess, glucocorticoid-remediable aldosteronism, renal artery stenosis and a deoxycorticosterone-producing tumour should be included in the differential diagnosis.^6^

**FIGURE 2:**
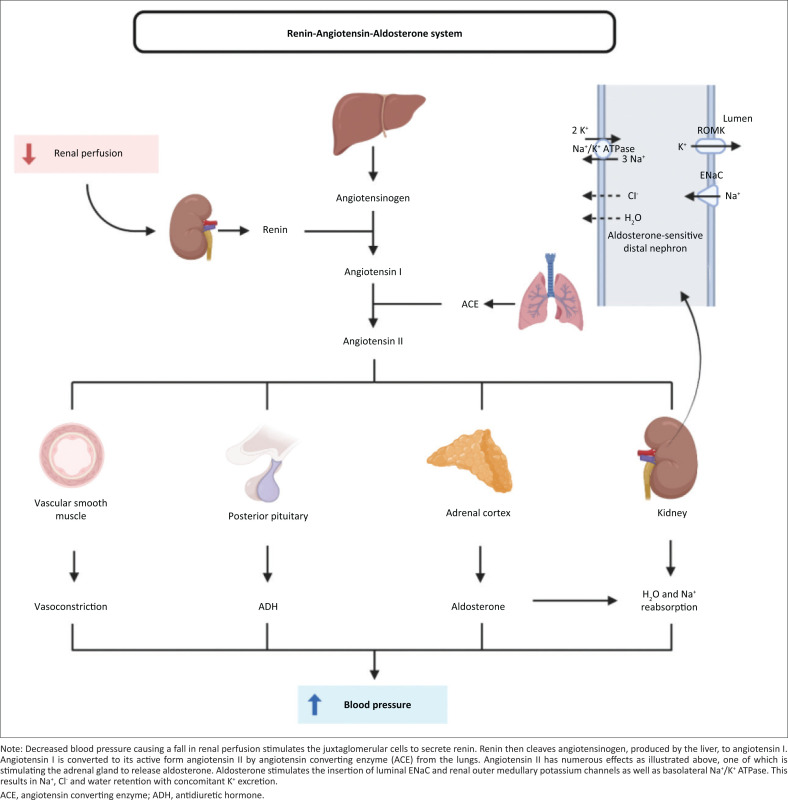
Renin-angiotensin aldosterone system physiology.

The presence of persistent hypertension with hypokalaemic metabolic alkalosis should raise the suspicion of unregulated ENaC activation.^4^ Sodium reabsorption in the epithelial cells of the distal renal tubule is regulated by the ENaC, which is activated through the renin-angiotensin-aldosterone system^6^ (Figure 2). It is important to determine if the hypertension is associated with low renin levels. In this instance, both serum aldosterone (< 27.0 pmol/L [RI: 49–643 pmol/L, supine], measured by Diagnostic Products Corporation Aldosterone Coat-a-count Kit, Diagnostic Products Corporation, Los Angeles, California, United States) and plasma renin concentration (0.5 mIU/L [RI: 6.5–36.2 mIU/L, supine], measured by CIS BIO Active Renin assay, Cisbio Bioassays, Codolet, France) levels were suppressed.

When the ENaC is activated independently of aldosterone stimulation, treatment with aldosterone antagonists has no effect. However, the use of amiloride or triamterene can lead to complete resolution of symptoms, as they are direct antagonists of the renal tubular ENaC and cause natriuresis while being both potassium and magnesium sparing.^3^ If there is a suspicion of an ENaC mutation based on the response to these drugs,^3^ whole exome sequencing should be undertaken.

The very rare missense single nucleotide variant (for which our patient is heterozygous), that causes the substitution of glycine to valine (p.Gly442Val), has been linked to hypertension with increased ENaC activity.^7,8^ The effect of this polymorphism has been assessed by measuring the urine-aldosterone to -potassium ratio.^7^ Increased ENaC activity would decrease this ratio as excess sodium absorption results in reduced aldosterone production and elevated urinary potassium excretion.^7^ This phenomenon has been confirmed in Liddle syndrome patients where the urine-aldosterone to -potassium ratio was lower in subjects with the polymorphism than in normal subjects.^9^ The association of the alpha ENaC polymorphism (for which our patient is heterozygous), that causes the substitution of alanine to threonine (pAla334Thr), has been related to hypertension in certain studies^10^ and is associated with increased ENaC activity of 1.6-fold^10^ in functional studies. These findings in the current patient indicate that the severe clinical phenotype observed could be attributed to the compound heterozygous mutations located at different subunits of the ENaC and may indicate the presence of a yet unrecognised pathological variant.

Analogous to disorders caused by mineralocorticoid excess, Liddle syndrome classically presents with hypertension, hypokalaemia and metabolic alkalosis. These findings are not always present, which may lead to under-diagnosis of the syndrome.^11^ Identification of this condition is challenging, as the differential diagnosis for secondary hypertension is broad and the syndrome may present atypically. Patients may have marked variations in phenotype, even with the same genotype.^3^ Liddle syndrome principally arises from a transport impairment causing increased sodium reabsorption and excretion of potassium and hydrogen ions in the distal renal tubule. Invariably, this leads to hypertension due to sodium and fluid retention with consequent hypokalaemic metabolic alkalosis and the suppression of renin and aldosterone through negative feedback.

Rare variants may, autonomously or cumulatively, cause hereditary disorders. Liu and colleagues observed substantial differences in serum potassium levels and symptom onset in rare and non-rare *SCNN1B* and *SCNN1G* variant carriers, suggesting potential pathogenicity of some variants.^12^ To date, approximately 31 different Liddle syndrome-causing alleles have been described in 72 families from four continents.^3,10,13^

Interpretation of results from next-generation sequencing technologies are challenging as they have increased not only the diagnostic sensitivity, but also the number of variants with uncertain clinical significance. It is feasible that additional mutations that increase ENaC activity and result in phenotypical Liddle syndrome will be discovered. In a study including patients with the Liddle syndrome phenotype from Kenya, Nigeria and South Africa, most patients had variants of a number of diverse genes that affect the ENaC channel.^13^ The authors speculated that certain patients may have combinations of variants that predispose to both increased aldosterone secretion and increased activity of the ENaC.^13^

In the assessment of patients with hyporeninaemic hypertension, investigation for a mutation in the ENaC subunits is recommended since early diagnosis and correct management of these patients may improve outcomes. Delayed treatment is associated with failure to thrive and hypertension-related morbidity and mortality from cardiovascular disease, cerebrovascular disease, nephrosclerosis and progressive renal failure. Furthermore, mutation studies permit clinicians to advocate for family screening based on the proband to identify carriers.^14^

This is the first case where a combined effect from mutations resulted in severe disease. Genetic testing (including whole exome sequencing) should be performed, when possible, to identify mutations in patients with suspected secondary hypertension and unusual presentation.^14^

### Conclusion

Liddle syndrome is a poorly understood, but treatable genetic disease. Mis- or late diagnosis may lead to unfavourable clinical sequelae. Thus, any infant presenting with hypertension and metabolic alkalosis, with or without hypokalaemia, should raise suspicion for Liddle syndrome, even in patients without a family history of hypertension. More functional studies are needed to characterise the numerous variants associated with the syndrome and their potential pathogenicity.
